# A comparison principle for doubly nonlinear parabolic partial differential equations

**DOI:** 10.1007/s10231-023-01381-4

**Published:** 2023-09-22

**Authors:** Verena Bögelein, Michael Strunk

**Affiliations:** Fachbereich Mathematik, Universität Salzburg, Hellbrunner Str. 34, 5020 Salzburg, Austria

**Keywords:** Doubly nonlinear parabolic PDE, Comparison principle, 35K55, 35K65, 35K67, 35A02

## Abstract

In this paper, we derive a comparison principle for non-negative weak sub- and super-solutions to doubly nonlinear parabolic partial differential equations whose prototype is

$$\begin{aligned} \partial _t u^q - {{\,\textrm{div}\,}}{\big (|\nabla u|^{p-2}\nabla u \big )}=0 \qquad \text{ in } \Omega _T, \end{aligned}$$

with $q>0$ and $p>1$ and $\Omega _T:=\Omega \times (0,T)\subset \mathbb {R}^{n+1}$. Instead of requiring a lower bound for the sub- or super-solutions in the whole domain $\Omega _T$, we only assume the lateral boundary data to be strictly positive. The main results yield some applications. Firstly, we obtain uniqueness of non-negative weak solutions to the associated Cauchy–Dirichlet problem. Secondly, we prove that any weak solution is also a viscosity solution.

## Introduction and main results

The parabolic partial differential equation

1.1 $$\begin{aligned} \partial _t u^q - {{\,\textrm{div}\,}}{\big (|\nabla u |^{p-2}\nabla u \big )}=0 \qquad \text{ in } \Omega _T, \end{aligned}$$

with some arbitrary exponents $q>0$ and $p>1$ is a non-trivial generalization of some well-studied problems. Here and in the following $\Omega _T=\Omega \times (0,T)$ denotes a space-time cylinder over a bounded domain $\Omega \subset \mathbb {R}^n$ and $T>0$. In its general form, (1.1) is called a doubly nonlinear pde. Only for the specific choice $q=1$ and $p=2$, it is linear and yields the heat equation. If $q=p-1$, it is homogeneous with respect to multiplication. The resulting pde is sometimes called Trudinger’s equation. In the case $p=2$, we obtain the porous medium equation, whereas the case $q=1$ yields the parabolic *p*-Laplace equation.

Properties of weak solutions to the porous medium equation and the parabolic *p*-Laplace equation are by now better understood than for the general doubly nonlinear pde (1.1). In this paper, we will investigate comparison principles for weak sub- and super-solutions to (1.1) as well as generalizations of (1.1). Roughly speaking, the comparison principle states that a sub-solution *u* and a super-solution *v* which satisfy $u\le v$ on the parabolic boundary $\partial _p\Omega _T=({\overline{\Omega }}\times \{0\})\cup (\partial \Omega \times (0,T))$ of the domain, must have the same property in the whole domain $\Omega _T$. Although it is generally understood to be a rather simple property, the comparison principle for doubly nonlinear equations is still far from being understood, and only special cases could be treated so far. The difficulties occur due to the lack of a weak time derivative and in particular in points where the solution is close to zero. Note that these difficulties do not occur for parabolic *p*-Laplace type equations, i.e., in the case $q=1$, in which the comparison principle can be shown by standard methods. Moreover, comparison principles for the prototype porous medium equation are presented in [30]. For more general equation of porous medium type, the situation is less clear.

In [2], Bamberger proved a comparison principle for weak solutions to doubly nonlinear equations under the additional assumption $\partial _t u^q, \partial _t v^q \in L^1(\Omega _T)$. In a similar spirit, Alt and Luckhaus [1] obtained a comparison principle for weak sub- and super-solutions, provided that $(\partial _t u^q - \partial _t v^q) \in L^1(\Omega _T)$. Also, the result of Diaz [12] requires an additional assumption on the time derivative. Unfortunately, these assumptions are quite restrictive, since they are not inherent in the definition of weak solution and in general not easy to verify.

Otto followed a different approach in [27]. He proved a comparison principle for weak sub- and super-solutions whose lateral boundary data are time independent. In particular, he avoided any extra regularity assumption on the sub- and super-solutions. Yet another approach was chosen by Ivanov, Mkrtychan, and Jäger in [20] for the case $q\in (0,1]$ and $p\in (1,2)$. Note that the parameter $\ell$ in [20] corresponds to $\frac{(1-q)(p-1)}{q}$ in (1.1). They allow time-dependent boundary data and prove a comparison principle for bounded and strictly positive sub- and super-solutions, i.e., the infimum of *u* and *v* on $\Omega _T$ is assumed to be strictly positive. Subsequently, Ivanov [18] extended the result to the range of exponents $q\in (0,1]$ and $p>1$. A similar result for Trudinger’s equation, i.e., the case $p>1$ and $q=p-1$ was established by Lindgren and Lindqvist in [26].

Our aim in this paper is to treat the full range of exponents $q>0$ and $p>1$. Moreover, we are able to weaken the infimum assumption. Instead of requiring the infimum of the sub- and super-solution to be strictly positive, we only assume the lateral boundary data of the super-solution to be strictly positive. Postponing a formal definition of weak sub/super-solutions to Sect. 2.2, our first main result is the following.

### Theorem 1.1

Let $q>0$, $p>1$ and suppose that *u* is a non-negative weak sub-solution and *v* a non-negative weak super-solution of (1.1) in $\Omega _T$ satisfying

1.2 $$\begin{aligned} \mathop {\mathrm {ess\,inf}}\limits \limits _{\partial \Omega \times (0,T)}v>0 \qquad \text{ and } \qquad \mathop {\mathrm {ess\,sup}}\limits \limits _{\partial \Omega \times (0,T)}u<\infty \text{ if } q>1. \end{aligned}$$

If

1.3 $$\begin{aligned} u\le v \quad \text{ on } \partial \Omega \times (0,T), \end{aligned}$$

then the following inequality holds

1.4 $$\begin{aligned} \displaystyle \int _{\Omega \times \{t_2\}} \left( u^q - v^q \right) _+ \textrm{d}x\le \displaystyle \int _{\Omega \times \{t_1\}} \left( u^q - v^q \right) _+ \textrm{d}x \end{aligned}$$

for every $$0\le t_1<t_2\le T$$.

As usual, the assumption $u\le v$ on $\partial \Omega \times (0,T)$ has to be understood in the sense that $(u-v)_+\in L^p(0,T;W^{1,p}_0(\Omega ))$. Applying Theorem 1.1 in the special situation where additionally $u(\cdot ,0)\le v(\cdot ,0)$ a.e. in $\Omega$ yields a comparison principle on parabolic cylinders.

### Theorem 1.2

Let $q>0$, $p>1$ and *u* be a non-negative weak sub-solution and *v* a non-negative weak super-solution of (1.1) in $\Omega _T$ satisfying (1.2). If

$$\begin{aligned} u\le v \qquad \text{ on } \partial _p \Omega _T, \end{aligned}$$

then we have

$$\begin{aligned} u\le v\qquad \text{ a.e. } \text{ in } \Omega _T. \end{aligned}$$

The approach in this paper is inspired by the proofs given in [18, 20, 26]. As mentioned above, the assumed lower bound of either the weak sub-solution or the weak super-solution in the whole of $\Omega _T$ is a strong restriction one would like to relinquish. In this paper, we were able to relax this condition to a lower bound on the lateral boundary. This has been achieved with the two expedient Lemmas 2.4 and 3.1. The first one allows to replace the sub-solution by another sub-solution which is bounded from below by a positive constant, as well as to replace the super-solution by a bounded super-solution. Assumption (1.2) ensures that the condition on the lateral boundary data is not violated. The difficulty in the proof of the comparison principle is firstly to choose a test-function which is regular enough. As we do not impose any assumption on the time derivatives, the choice of test-function is a delicate issue, in particular when $q\not =1$. Therefore, a suitable mollification is necessary. Secondly, without a lower bound on the weak sub/super-solution in $\Omega _T$, we somehow have to work around this assumption by determining at least suitable boundary conditions. The latter allows us to apply Lemma 2.4 in order to construct an auxiliary sub-solution which is on the one hand strictly positive in $\Omega _T$ and on the other hand smaller than the super-solution on the lateral boundary of $\Omega _T$. This is achieved by working with $\displaystyle \max \{u,\kappa \}$, for a suitable constant $\kappa >0$, instead of *u*, where *u* denotes the weak sub-solution. Similarly, in the case $q>1$ we also make use of Lemma 2.4 in order to replace the weak super-solution *v* by the auxiliary super-solution $\displaystyle \min \{v,M\}$ for appropriate *M* large enough. We emphasize that no upper bound of weak sub-solutions on the lateral boundary is necessary, except in the case $q>1$. This is achieved with the help of Lemma 3.1 that also has been used in [20]. The application of Lemma 3.1 allows to avoid a time mollification such as Steklov average or exponential mollification in the test-function. Note that the case $q=1$, which yields the parabolic *p*-Laplace equation, is easier and neither a lower nor an upper bound for the lateral boundary data is needed. Since this is classical, we do not go into further detail.

For particular ranges of exponents *q* and *p*, we obtain stronger results in a local setting. If either $0<q\le p-1$, or $0<p-1<q<\frac{n(p-1)+p}{(n-p)_+}$, then weak sub-solutions to (1.1) are locally bounded. This property is exploited in Corollary 3.4 below. A further restriction of the exponents to the range $0<p-1 \le q < \frac{n(p-1)}{(n-p)_+}$ even allows to prove in a local setting a comparison principle for weak solutions without any additional assumptions like upper or lower bounds.

### Theorem 1.3

Let $0<p-1 \le q < \frac{n(p-1)}{(n-p)_+}$ and *u*, *v* be non-negative local weak solutions of (1.1) in $\Omega _T$. Further, let $K \Subset \Omega$ and $0<t_1<t_2<T$. If

$$\begin{aligned} u\le v \qquad \text{ on } \partial _p \big (K\times (t_1,t_2)\big ), \end{aligned}$$

then we have

$$\begin{aligned} u\le v\qquad \text{ in } K\times (t_1,t_2). \end{aligned}$$

Note that we can also allow $t_1=0$ if *u* and *v* are defined until the initial time $t=0$. The key ingredient to the proof of Theorem 1.3 is a Harnack inequality which ensures that non-negative local weak solutions of (1.1) are either zero or strictly positive on any time slice.

Naturally, the interest in a comparison principle for (1.1) with a nonzero right-hand side *f* arises. Thus, instead of (1.1), one could rather consider its inhomogeneous version

1.5 $$\begin{aligned} \partial _t u^q - \Delta _p u = f\qquad \text{ in } \Omega _T. \end{aligned}$$

We obtain similar comparison principles for the preceding equation by slightly adapting the proofs of the main results in Theorems 1.1 and 1.2, provided *f* belongs to a suitable parabolic Lebesgue space; see Definition 4.1 below. A further generalization concerns the vector field in the diffusion part of (1.1). Instead of the pure *p*-Laplace operator, our results continue to hold for vector fields of the form

$$\begin{aligned} A(x,t,u,\xi ):\Omega _T\times \mathbb {R}_+\times \mathbb {R}^n \rightarrow \mathbb {R}^n \end{aligned}$$

and the associated doubly nonlinear differential equation

1.6 $$\begin{aligned} \partial _t u^q - {{\,\textrm{div}\,}}A(x,t,u,\nabla u) = f \qquad \text{ in } \Omega _T. \end{aligned}$$

Here, we assume *A* to be a Carathéodory function which satisfies suitable *p*-growth, Lipschitz and monotonicity conditions; see the set of assumptions (4.5). We obtain similar comparison principles also for (1.6). However, in contrast to the comparison principle derived for the prototype equation, the proof in the general setting requires more care and a careful use of the assumed monotonicity and Lipschitz conditions is required. Since our results for both equations (1.5) and (1.6) are similar to those for the model equation (1.1), we only state the latter here.

Finally, we note that also the comparison principles shown in [18, 20] apply to more general doubly nonlinear partial differential equations than the prototype one (1.1). To obtain the addressed pde in [18, 20], one may substitute $v = u^q$ in (1.1) to derive the equivalent form

1.7 $$\begin{aligned} \partial _t v - {{\,\textrm{div}\,}}{\left( q^{1-p}v^{\frac{(1-q)(p-1)}{q}}\left| \nabla v\right| ^{p-2}\nabla v\right) }=0\qquad \text{ in } \Omega _T, \end{aligned}$$

for $q>0$ and $p>1$. The preceding presentation illustrates the correspondence $\ell = \frac{(1-q)(p-1)}{q}$. Therefore, the assumption $\ell \ge 0$ in [18, 20] corresponds to $q\in (0,1]$ in (1.1).

**Plan of the paper.** Firstly, in Sect. 2 we will introduce the setting and notations we are working with, including the definition of (non-negative) weak (sub-/super-)solutions to (1.1). We also define the two auxiliary functions $H_\delta$ and $G_\delta$, $\delta >0$, used in the proof of the comparison principle in Theorem 1.1. Additionally, we introduce two different mollifications in time, namely the Steklov-average and the exponential mollification.

Section 3 contains the main part of the paper, where the comparison principles from Theorems 1.1 and 1.2 are proved. Respective results for the local setting are given in Subsection 3.2, where the comparison principle from Theorem 1.3 is shown. We will then, in Sect. 4, discuss possible generalizations of the comparison principle to inhomogeneous doubly nonlinear equations and more general vector fields. In Sect. 5, we provide uniqueness results for Cauchy–Dirichlet problems associated with a doubly nonlinear equation, which are a direct consequence of the comparison principles obtained before.

Finally, in Sect. 6 we will show as application of the comparison principle that every weak solution of (1.1) is also a viscosity solution in the sense of [11]. In particular, this result implies existence of viscosity solutions.

## Preliminaries

### Notation

Throughout $\Omega _T = \Omega \times (0,T)$ denotes a space-time cylinder, where $\Omega \subset \mathbb {R}^n$ is a bounded domain and (0, *T*) represents a time interval for a certain time $T>0$. The parabolic boundary of $\Omega _T$ will be denoted by

$$\begin{aligned} \partial _p \Omega _T = \big ({\overline{\Omega }}\times \{0\}\big ) \cup \big (\partial \Omega \times (0,T)\big ). \end{aligned}$$

For a function $f\in L^1(\Omega _T)\cong L^1(0,T;L^1(\Omega ))$, we also write *f*(*t*) instead of $f(\cdot ,t)$ whenever it is convenient. Moreover, we will abbreviate the *p*-Laplace operator by

2.1 $$\begin{aligned} \Delta _p u :={{\,\textrm{div}\,}}{\big (|\nabla u|^{p-2}\nabla u \big )}. \end{aligned}$$

Throughout the paper, we will not distinguish between the Euclidean norm $\Vert \cdot \Vert$ in $\mathbb {R}^n$ for $n\ge 2$ and the absolute value $|\cdot |$ in $\mathbb {R}$. Both shall be denoted by $|\cdot |$ and the meaning will be clear from the context. For matrices $X\in \mathbb {R}^{n\times n}$, we will always use the spectral norm given by $\left\| X \right\| = \sqrt{\lambda _{\max }}$, where $\lambda _{\max }$ denotes the largest eigenvalue of $X^{\top } X$. Recall that the spectral norm is consistent with the Euclidean vector norm, that is

$$\begin{aligned} \left| Xv\right| \le \left\| X\right\| \left| v\right| \qquad \text{ for } \text{ any } v\in \mathbb {R}^n \text{ and } X\in \mathbb {R}^{n\times n}. \end{aligned}$$

Furthermore, the trace of a matrix $X\in \mathbb {R}^{n\times n}$ shall be expressed by ${{\,\textrm{Tr}\,}}(X)$.

The positive part of some quantity $a\in \mathbb {R}$ is denoted by $a_+ = \max \{a,0\}$, whereas the negative part by $a_-=\max \{-a,0\}$. Constants will always be denoted by *c* or $c(\cdot )$, where only the dependence of the constants is stated. However, constants may change from line to line without further explanation.

### Definition of weak solution

Although it is standard, we briefly state the definition of a (local) weak solution that we use throughout the paper.

#### Definition 2.1

(Weak solution) A non-negative measurable function $u:\Omega _T\rightarrow \mathbb {R}_{\ge 0}$ in the class

$$\begin{aligned} u\in C\big ([0,T];L^{q+1}(\Omega ) \big ) \cap L^{p}\big (0,T;W^{1,p}(\Omega )\big ) \end{aligned}$$

is a non-negative weak sub(super)-solution of (1.1) if

2.2 $$\begin{aligned} \iint _{\Omega _T}\big [-u^q\partial _t\phi + |\nabla u|^{p-2}\nabla u \cdot \nabla \phi \big ]\,\textrm{d}x\textrm{d}t\le (\ge )\, 0 \end{aligned}$$

for any non-negative function

$$\begin{aligned} \phi \in W^{1,q+1}_{0}\big (0,T;L^{q+1}(\Omega )\big ) \cap L^p\big (0,T;W^{1,p}_0(\Omega )\big ). \end{aligned}$$

A non-negative function *u* is a non-negative weak solution of (1.1) if it is both, a weak sub-solution and a weak super-solution.

#### Definition 2.2

(Local weak solution) A non-negative measurable function $u:\Omega _T\rightarrow \mathbb {R}_{\ge 0}$ in the class

$$\begin{aligned} u\in C\big (0,T;L^{q+1}_{\textrm{loc}}(\Omega ) \big )\cap L^{p}_{\textrm{loc}}\big (0,T;W^{1,p}_{\textrm{loc}}(\Omega )\big ) \end{aligned}$$

is a non-negative local weak sub(super)-solution of (1.1) if for every $K\Subset \Omega$ and every sub-interval $[t_1,t_2]\subset (0,T)$ we have

$$\begin{aligned} \int _{K}u^q \phi \,\textrm{d}x\,\Big |^{t_2}_{t_1} + \iint _{K\times (t_1,t_2)} \big [-u^q\partial _t\phi + \left| \nabla u\right| ^{p-2}\nabla u \cdot \nabla \phi \big ]\,\textrm{d}x\textrm{d}t\le (\ge )\, 0 \end{aligned}$$

for any non-negative function

$$\begin{aligned} \phi \in W^{1,q+1}_{\textrm{loc}}\left( 0,T;L^{q+1}(K)\right) \cap L^p_{\textrm{loc}}\big (0,T;W^{1,p}_0(K)\big ). \end{aligned}$$

A non-negative function *u* is a non-negative local weak solution of (1.1) if it is both, a local weak sub-solution and a local weak super-solution.

Existence of weak solutions to the Cauchy–Dirichlet problem associated with (1.1) has been shown in [1]. It is worth noticing that due to Definition 2.1 weak sub/super-solutions belong to the space

$$\begin{aligned} u\in C\big ([0,T];L^{q+1}(\Omega ) \big ) \cap L^{p}\big (0,T;W^{1,p}(\Omega )\big ) \end{aligned}$$

and thus, are assumed to be continuous functions in time. However, this is not restrictive as shown in [6, Proposition 4.9].

### Mollification in time

In view of their definition, weak solutions are not necessarily weakly differentiable with respect to the time variable. This difficulty is usually overcome by certain regularization procedures. We will work with two different mollifications. The first one is the Steklov-average, cf. [10]. For a function $f \in L^1(\Omega _T)$ and $0<h<T$, we define its *Steklov-average* $[f]_{ h}$ by

2.3 $$\begin{aligned}{}[f]_{h}(x,t):= \left\{ \begin{array}{cl} \displaystyle {\frac{1}{h} \int _{t}^{t+h} f(x,\tau ) \,\textrm{d}\tau ,} &{} t\in (0,T-h), \\ 0, &{} t\in [T-h,T) . \end{array} \right. \end{aligned}$$

Rewriting inequality (2.2) in terms of Steklov-means $[u]_h$ of *u*, yields

2.4 $$\begin{aligned} \int _{\Omega \times \{t\}}\Big [\partial _t [u^q]_h \phi + \big [|\nabla u|^{p-2}\nabla u\big ]_h \cdot \nabla \phi \Big ]\,\textrm{d}x\le (\ge )\, 0 \end{aligned}$$

for any non-negative function $\phi \in W^{1,p}_0(\Omega )$ and any $t\in (0,T)$.

In the course of the paper, we will also need another mollification in time. For any $f\in L^1(\Omega _T)$ and $h>0$, we introduce the exponential mollification

2.5 $$\begin{aligned} \llbracket f \rrbracket _h(x,t):= \frac{1}{h} \int _0^t \mathrm e^{\frac{\tau -t}{h}} f(x,\tau ) \, \textrm{d}\tau \quad \text {and}\quad \llbracket f \rrbracket _{\bar{h}}(x,t):= \frac{1}{h} \int _t^T \mathrm e^{\frac{t-\tau }{h}} f(x,\tau ) \, \textrm{d}\tau , \end{aligned}$$

as defined in [23].

### Auxiliary material

The following lemma that can be found in [14, Lemma 2.2] will be useful in order to deal with the nonlinearity of the differential equation.

#### Lemma 2.3

Let $k\in {\mathbb {N}}$. For any $\alpha >1$, there exists a constant $c=c(\alpha )$ such that

$$\begin{aligned} \frac{1}{c}\big ||a|^{\alpha -1}a - |b|^{\alpha -1}b\big | \le \big (|a|^{\alpha -1} + |b|^{\alpha -1}\big )|a-b| \le c \big ||a|^{\alpha -1}a - |b|^{\alpha -1}b\big | \end{aligned}$$

for all $$a,b\in \mathbb {R}^k$$.

Weak sub(super)-solutions preserve this property when taking the maximum, respectively, minimum, with a constant. For the proof of this fact, we proceed similar as in [7, Lemma A.1]. For the sake of completeness, we provide the details.

#### Lemma 2.4

Let $q>0$, $p>1$ and *u* be a non-negative weak sub-solution of (1.1) in the sense of Definition 2.1. Then, for any $\kappa >0$ the function $\max \{u,\kappa \}$ is also a weak sub-solution of (1.1).

Similarly, if *v* is a non-negative weak super-solution of (1.1), then for any $M>0$ also $\min \{v,M\}$ is a weak super-solution.

#### Proof

Only the sub-solution case is treated. The super-solution case may be treated in a similar way. For $h,\mu >0$ and $\eta \in C^1_0(\Omega _T)$ such that $\eta \ge 0$ in $\Omega _T$, we choose the test function

$$\begin{aligned} \phi = \eta \phi _h, \quad \text{ where } \phi _h:= \frac{(\llbracket u\rrbracket _{{\bar{h}}}-\kappa )_+}{(\llbracket u\rrbracket _{{\bar{h}}}-\kappa )_++\mu } \end{aligned}$$

in the weak form (2.2) of the differential equation, i.e.,

2.6 $$\begin{aligned} \iint _{\Omega _T}\big [-u^q\partial _t\phi + |\nabla u|^{p-2}\nabla u \cdot \nabla \phi \big ]\,\textrm{d}x\textrm{d}t\le \, 0. \end{aligned}$$

We start by considering the term involving the time derivative. We obtain

2.7 $$\begin{aligned}&-\iint _{\Omega _T} u^q\partial _t\phi \,\textrm{d}x\textrm{d}t\nonumber \\&\quad = \iint _{\Omega _T} \big (\llbracket u\rrbracket _{{\bar{h}}}^q - u^q\big )\partial _t\phi \,\textrm{d}x\textrm{d}t- \iint _{\Omega _T} \llbracket u\rrbracket _{{\bar{h}}}^q \partial _t\phi \,\textrm{d}x\textrm{d}t\nonumber \\&\quad = \iint _{\Omega _T} \eta \big (\llbracket u\rrbracket _{{\bar{h}}}^q - u^q\big ) \partial _t\phi _h \,\textrm{d}x\textrm{d}t\nonumber \\&\qquad + \iint _{\Omega _T} \partial _t \eta \big (\llbracket u\rrbracket _{{\bar{h}}}^q - u^q\big ) \phi _h\,\textrm{d}x\textrm{d}t+ \iint _{\Omega _T} \partial _t\llbracket u\rrbracket _{{\bar{h}}}^q \phi \,\textrm{d}x\textrm{d}t\nonumber \\&\quad = \text{ I } + \text{ II } + \text{ III }, \end{aligned}$$

with the obvious meaning of I – III. The first term on the right-hand side of (2.7) is non-negative, which can be seen by the following computation

$$\begin{aligned} \text{ I }&= \mu \iint _{\Omega _T} \eta \big (\llbracket u\rrbracket _{{\bar{h}}}^q - u^q\big ) \frac{\partial _t(\llbracket u\rrbracket _{{\bar{h}}}-\kappa )_+}{\big [(\llbracket u\rrbracket _{{\bar{h}}}-\kappa )_++\mu \big ]^2} \,\textrm{d}x\textrm{d}t\\&= \frac{\mu }{h} \iint _{\Omega _T} \eta \big (\llbracket u\rrbracket _{{\bar{h}}}^q - u^q\big ) \big (\llbracket u\rrbracket _{{\bar{h}}} - u\big ) \frac{\chi _{\{\llbracket u\rrbracket _{{\bar{h}}}>\kappa \}}}{\big [(\llbracket u\rrbracket _{{\bar{h}}}-\kappa )_++\mu \big ]^2} \,\textrm{d}x\textrm{d}t\ge 0. \end{aligned}$$

From the second to last line, we used the identity $\partial _t \llbracket u\rrbracket _{{\bar{h}}}=\frac{1}{h} (\llbracket u\rrbracket _{{\bar{h}}} - u)$. Now, we turn our attention to the third term in (2.7).To this aim, we define

$$\begin{aligned} f(u,\kappa ,\mu ) := \kappa ^q + q \displaystyle \int _{\kappa }^{u}\frac{s^{q-1}(s-\kappa )_+}{(s-\kappa )_++\mu }\,\textrm{d}s. \end{aligned}$$

In view of the chain rule, it is easy to see that

$$\begin{aligned} \partial _t f\big (\llbracket u\rrbracket _{{\bar{h}}},\kappa ,\mu \big ) = \partial _t \llbracket u\rrbracket _{{\bar{h}}}^q \frac{(\llbracket u\rrbracket _{{\bar{h}}}-\kappa )_+}{(\llbracket u\rrbracket _{{\bar{h}}}-\kappa )_++\mu } = \partial _t \llbracket u\rrbracket _{{\bar{h}}}^q \phi _h. \end{aligned}$$

Using the previous computation and integrating by parts yields

$$\begin{aligned} \text{ III }&= \iint _{\Omega _T} \eta \partial _t\llbracket u\rrbracket _{{\bar{h}}}^q \phi _h \,\textrm{d}x\textrm{d}t= \iint _{\Omega _T}\eta \partial _t f\big (\llbracket u\rrbracket _{{\bar{h}}},\kappa ,\mu \big )\,\textrm{d}x\textrm{d}t\\&= -\iint _{\Omega _T} \partial _t \eta f\big (\llbracket u\rrbracket _{{\bar{h}}},\kappa ,\mu \big )\,\textrm{d}x\textrm{d}t. \end{aligned}$$

Inserting these informations into (2.7), we obtain

$$\begin{aligned} -\iint _{\Omega _T} u^q\partial _t\phi \,\textrm{d}x\textrm{d}t\ge \iint _{\Omega _T} \partial _t \eta \big (\llbracket u\rrbracket _{{\bar{h}}}^q - u^q\big ) \phi _h\,\textrm{d}x\textrm{d}t- \iint _{\Omega _T} \partial _t \eta f\big (\llbracket u\rrbracket _{{\bar{h}}},\kappa ,\mu \big )\,\textrm{d}x\textrm{d}t. \end{aligned}$$

The first term on the right-hand side vanishes in the limit $h\downarrow 0$. Therefore, inserting this inequality into (2.6) and then, letting $h\downarrow 0$, we arrive at

$$\begin{aligned} -\iint _{\Omega _T} \partial _t\eta f(u,\kappa ,\mu )\,\textrm{d}x\textrm{d}t+ \iint _{\Omega _T} |\nabla u|^{p-2}\nabla u \cdot \nabla \bigg [\eta \frac{(u-\kappa )_+}{(u-\kappa )_++\mu }\bigg ]\,\textrm{d}x\textrm{d}t\le \, 0 . \end{aligned}$$

Next, we will treat the diffusion term, i.e., the second term on the left hand side of the preceding inequality. We have

$$\begin{aligned} \iint _{\Omega _T}&|\nabla u|^{p-2}\nabla u \cdot \nabla \bigg [\eta \frac{(u-\kappa )_+}{(u-\kappa )_++\mu }\bigg ]\,\textrm{d}x\textrm{d}t\\&= \iint _{\Omega _T}\bigg [|\nabla u|^{p-2}\nabla u\cdot \nabla \eta \frac{(u-\kappa )_+}{(u-\kappa )_++\mu } + |\nabla u|^{p-2} \nabla u\cdot \frac{\eta \mu \nabla (u-\kappa )_+}{[(u-\kappa )_++\mu ]^2}\bigg ] \,\textrm{d}x\textrm{d}t\\&\ge \iint _{\Omega _T}|\nabla u|^{p-2}\nabla u\cdot \nabla \eta \frac{(u-\kappa )_+}{(u-\kappa )_++\mu } \,\textrm{d}x\textrm{d}t. \end{aligned}$$

Inserting this above yields

$$\begin{aligned} -\iint _{\Omega _T} \partial _t\eta f(u,\kappa ,\mu )\,\textrm{d}x\textrm{d}t+ \iint _{\Omega _T}|\nabla u|^{p-2}\nabla u\cdot \nabla \eta \frac{(u-\kappa )^+}{(u-\kappa )^++\mu } \,\textrm{d}x\textrm{d}t\le 0. \end{aligned}$$

A direct calculation shows that

$$\begin{aligned} \lim \limits _{\mu \downarrow 0}f(u,\kappa ,\mu ) = \max \{u,\kappa \}^q \end{aligned}$$

and

$$\begin{aligned} \lim \limits _{\mu \downarrow 0}\frac{(u-\kappa )^+}{(u-\kappa )^++\mu } = \chi _{\{u>\kappa \}}. \end{aligned}$$

Furthermore, note that $\chi _{\{u>\kappa \}}|\nabla u|^{p-2}\nabla u = |\nabla \max \{u,\kappa \}|^{p-2}\nabla \max \{u,\kappa \}$. Therefore, letting $\mu \downarrow 0$ and using an approximating argument in order to obtain the desired inequality for an arbitrary test function

$$\begin{aligned} \phi \in W^{q+1}_{0}\left( 0,T;L^{q+1}(\Omega )\right) \cap L^p\big (0,T;W^{1,p}_0(\Omega )\big ) \end{aligned}$$

yields

$$\begin{aligned} \iint _{\Omega _T}\big [-\max \{u,\kappa \}^q\partial _t\phi + \left| \nabla \max \{u,\kappa \}\right| ^{p-2}\nabla \max \{u,\kappa \} \cdot \nabla \phi \big ]\,\textrm{d}x\textrm{d}t\le \, 0, \end{aligned}$$

proving that $\max \{u,\kappa \}$ is a weak sub-solution of (1.1). $\square$

## Comparison principles

Our aim in this section is to prove the comparison principles for the doubly nonlinear equation (1.1). We first turn our attention to weak sub- and super-solutions in $\Omega _T$ and subsequently consider the local setting.

### Comparison principles in a global setting

In this subsection, we will accomplish the proofs of Theorems 1.1 and 1.2. The main difficulty stems from the nonlinearity appearing in the time derivative part of (1.1). As illustrated for the homogeneous equation, i.e., the case $q=p-1$ in [26,  (3.1)], a comparison principle can be derived quite easily if the weak time derivative of $u^q$ exists. However, such a property is not implemented in the definition of a weak solution. Without existence of a weak time derivative, the test function has to be chosen very carefully and certain approximation arguments are needed.

Throughout the proof, we shall use the following two auxiliary functions. The first one is a piecewise affine approximation of the indicator function

3.1 $$\begin{aligned} H_\delta (x) := \displaystyle {\left\{ \begin{array}{ll} 1, &{} \quad x\ge \delta ,\\ \frac{x}{\delta }, &{} \quad 0< x < \delta ,\\ 0, &{} \quad x\le 0 \end{array}\right. } \end{aligned}$$

for $\delta >0$. The second one is its primitive and approximates the positive part

$$\begin{aligned} G_\delta (x) := \displaystyle {\left\{ \begin{array}{ll} x - \frac{\delta }{2}, &{} \quad x\ge \delta ,\\ \frac{x^2}{2\delta }, &{} \quad 0< x < \delta ,\\ 0, &{} \quad x\le 0. \end{array}\right. } \end{aligned}$$

Note that $G_\delta '(x) = H_\delta (x)$ for any $x\in \mathbb {R}$. The inequality stated in the next Lemma was already used in the proof of [20, Proposition 2.1]. It allows to choose a test function without dependency on any mollifiers like Steklov-average or exponential mollification in the proof of the comparison principle.

#### Lemma 3.1

Let $\delta >0$ and $f\in C(0,T;L^1(\Omega ))$. Then, for any $0<h<T$ the following inequality holds

3.2 $$\begin{aligned} \partial _t [G_\delta (f)]_{h} \le \partial _t [f]_{h} H_\delta (f) \qquad \text{ a.e. } \text{ in } \Omega _T \end{aligned}$$

#### Proof

For $t\in [T-h,T)$ inequality, (3.2) is trivial. Therefore, it remains to consider $t\in (0,T-h)$. The definition of the Steklov-average in (2.3) yields

$$\begin{aligned} \partial _t [G_\delta (f)]_{h}(t)&= \tfrac{1}{h} \big [G_\delta \left( f(t+h)\right) - G_\delta (f(t))\big ] \end{aligned}$$

and

$$\begin{aligned} \partial _t [f]_{h}&= \tfrac{1}{h} \big [f(t+h) - f(t)\big ]. \end{aligned}$$

Thus, inequality (3.2) simplifies to

$$\begin{aligned} G_\delta \left( f(t+h)\right) - G_\delta \left( f(t)\right) \le \big (f(t+h) - f(t) \big ) H_\delta \left( f(t)\right) . \end{aligned}$$

In view of the convexity of the mapping $\mathbb {R}\ni x \mapsto G_\delta (x)$, we have

$$\begin{aligned} G_\delta (y) - G_\delta (x) \ge G'_\delta (x)(y-x)\qquad \text{ for } \text{ any } x,y\in \mathbb {R}. \end{aligned}$$

Thus, setting $y = f(t+h)$ and $x = f(t)$ yields the desired inequality. $\square$

We start with the following preliminary version of the comparison principle, where we additionally require either the sub- or the super-solution to be bounded from above and below by positive constants.

#### Proposition 3.2

Let $q>0$, $p>1$ and *u* be a non-negative weak sub-solution and *v* a non-negative weak super-solution of (1.1) in $\Omega _T$. Suppose that either

3.3 $$\begin{aligned} u\ge \epsilon \quad \text{ or }\quad v\ge \epsilon \quad \text{ a.e. } \text{ in } \Omega _T \end{aligned}$$

for some $\epsilon >0$ and in the case $q> 1$ assume furthermore that either *u* or *v* is bounded. If

$$\begin{aligned} u\le v \quad \text{ on } \partial \Omega \times (0,T), \end{aligned}$$

then the following inequality holds

3.4 $$\begin{aligned} \displaystyle \int _{\Omega \times \{t_2\}} \left( u^q - v^q \right) _+ \,\textrm{d}x\le \displaystyle \int _{\Omega \times \{t_1\}} \left( u^q - v^q \right) _+ \,\textrm{d}x \end{aligned}$$

for every $$0\le t_1<t_2\le T$$.

#### Proof

For $h\in (0,T)$, we consider the Steklov formulation (2.4) of (2.2) for *u* and *v*. Adding both inequalities yields

$$\begin{aligned} \int _{\Omega \times \{t\}} \partial _t[u^q - v^q]_h \phi \,\textrm{d}x\le \int _{\Omega \times \{t\}}\left[ |\nabla v|^{p-2}\nabla v -|\nabla u|^{p-2}\nabla u \right] _h\cdot \nabla \phi \,\textrm{d}x\end{aligned}$$

for any $\phi \in W^{1,p}_0(\Omega )$ and any $t\in (0,T)$. Note that a weak time derivative for the test functions is not needed in this formulation. We now integrate this inequality with respect to $t \in (t_1,t_2)\subset (0,T)$ and choose the test-function $\phi =H_\delta (u^q -v^q)$ with $0<\delta \le \min \{1,\frac{\epsilon ^q}{2}\}$, which is admissible since $u^q\le v^q$ on the lateral boundary $\partial \Omega \times (0,T)$. Recall that $H_\delta$ is defined in (3.1). In this way, we obtain

3.5 $$\begin{aligned} \iint _{\Omega \times (t_1,t_2)}&\partial _t [u^q - v^q]_{h} H_\delta (u^q -v^q )\,\textrm{d}x\textrm{d}t\nonumber \\&\le \iint _{\Omega \times (t_1,t_2)} \left[ |\nabla v|^{p-2}\nabla v -|\nabla u|^{p-2}\nabla u \right] _{h}\cdot \nabla H_\delta ( u^q - v^q ) \,\textrm{d}x\textrm{d}t. \end{aligned}$$

Applying Lemma 3.1 with $f=u^q -v^q$ to the integrand on the left-hand side, we find

3.6 $$\begin{aligned} \iint _{\Omega \times (t_1,t_2)}&\partial _t [ G_\delta (u^q - v^q)]_{h} \,\textrm{d}x\textrm{d}t\nonumber \\&\le \iint _{\Omega \times (t_1,t_2)} \left[ |\nabla v|^{p-2}\nabla v -|\nabla u|^{p-2}\nabla u \right] _{h}\cdot \nabla H_\delta ( u^q - v^q ) \,\textrm{d}x\textrm{d}t. \end{aligned}$$

We now focus on the integral on the left-hand side of (3.6). Letting $h\downarrow 0$, we obtain

$$\begin{aligned} \lim _{h \downarrow 0 }\iint _{\Omega \times (t_1,t_2)} \partial _t [G_\delta (u^q - v^q)]_{h} \,\textrm{d}x\textrm{d}t&= \lim _{h \downarrow 0 } \int _{\Omega } [G_\delta (u^q- v^q)]_h \,\textrm{d}x\bigg |^{t_2}_{t_1} \\&= \int _{\Omega } G_\delta (u^q- v^q) \,\textrm{d}x\bigg |^{t_2}_{t_1}. \end{aligned}$$

Next, we justify the passage to the limit $h\downarrow 0$ for the integral on the right-hand side of (3.6). A direct computation yields

$$\begin{aligned} \iint _{\Omega \times (t_1,t_2)}&\left[ |\nabla v|^{p-2}\nabla v -|\nabla u|^{p-2}\nabla u \right] _{h}\cdot \nabla H_\delta \left( u^q - v^q\right) \,\textrm{d}x\textrm{d}t\nonumber \\&= \frac{1}{\delta }\iint _{\Omega _{\delta }}\left[ |\nabla v|^{p-2}\nabla v -|\nabla u|^{p-2}\nabla u \right] _{h}\cdot \nabla (u^q - v^q) \,\textrm{d}x\textrm{d}t, \end{aligned}$$

where

$$\begin{aligned} \Omega _{\delta }:= \big \{(x,t)\in \Omega \times (t_1,t_2) :\, 0< u^q(x,t) - v^q(x,t) < \delta \big \}. \end{aligned}$$

Since

$$\begin{aligned} |\nabla v|^{p-2}\nabla v -|\nabla u|^{p-2}\nabla u \in L^{\frac{p}{p-1}}(\Omega _T), \end{aligned}$$

we have

$$\begin{aligned} \left[ |\nabla v|^{p-2}\nabla v -|\nabla u|^{p-2}\nabla u \right] _{h} \rightarrow |\nabla v|^{p-2}\nabla v -|\nabla u|^{p-2}\nabla u \quad \text{ in } L^{\frac{p}{p-1}}(\Omega _T) \end{aligned}$$

as $h\downarrow 0$. Our next aim is to ensure that $\nabla (u^q - v^q)\in L^p(\Omega _\delta )$. This will be a consequence of assumption (3.3) and the definition of $\Omega _\delta$. We first consider the case $0<q\le 1$. If $v\ge \epsilon$ in $\Omega _T$, then we have $u>\epsilon$ in $\Omega _\delta$. Otherwise, if $u\ge \epsilon$ in $\Omega _T$, then we have $v>2^{-\frac{1}{q}}\epsilon$ in $\Omega _\delta$ by the choice of $\delta$. In any case, we find that

$$\begin{aligned} |\nabla u^q| = q u^{q-1}|\nabla u| \le q \epsilon ^{q-1} |\nabla u| \quad \text{ in } \Omega _\delta \end{aligned}$$

and

$$\begin{aligned} |\nabla v^q| = q v^{q-1}|\nabla v| \le c(q) \epsilon ^{q-1} |\nabla v| \quad \text{ in } \Omega _\delta . \end{aligned}$$

On the other hand, in the case $q>1$ we assume that either *u* or *v* is bounded. Therefore, there exists a constant $M>0$ such that either $u\le M$ or $v\le M$ in $\Omega _T$. Since $\delta \le 1$, this implies $u^q<1+M^q$ and $v^q<1+M^q$ in $\Omega _\delta$, so that

$$\begin{aligned} |\nabla v^q| \le c(q,M)|\nabla v| \ \ \text{ in } \Omega _T \quad \text{ and }\quad |\nabla u^q| \le c(q,M)|\nabla u| \quad \text{ in } \Omega _\delta . \end{aligned}$$

Thus, we have shown in any case that $\nabla (u^q - v^q)\in L^p(\Omega _\delta )$ and therefore, we may pass to the limit $h\downarrow 0$ also on the right-hand side of (3.6) and derive

$$\begin{aligned} \lim _{h\downarrow 0}&\iint _{\Omega \times (t_1,t_2)} \left[ |\nabla v|^{p-2}\nabla v -|\nabla u|^{p-2}\nabla u \right] _{h}\cdot \nabla H_\delta ( u^q - v^q ) \,\textrm{d}x\textrm{d}t\\&= \frac{1}{\delta } \iint _{\Omega _{\delta }}\left( |\nabla v|^{p-2}\nabla v -|\nabla u|^{p-2}\nabla u \right) \cdot \nabla (u^q - v^q) \,\textrm{d}x\textrm{d}t. \end{aligned}$$

In conclusion, after passing to the limit $h\downarrow 0$ on both sides of (3.6), we obtain

3.7 $$\begin{aligned} \int _{\Omega \times \{t_2\}}&G_\delta \left( u^q - v^q \right) \,\textrm{d}x- \int _{\Omega \times \{t_1\}} G_\delta \left( u^q - v^q \right) \,\textrm{d}x \nonumber \\&\le \frac{1}{\delta } \iint _{\Omega _{\delta }}\left( |\nabla v|^{p-2}\nabla v -|\nabla u|^{p-2}\nabla u \right) \cdot \nabla (u^q - v^q) \,\textrm{d}x\textrm{d}t. \end{aligned}$$

A simple calculation yields the identity

3.8 $$\begin{aligned} \nabla (u^q - v^q) = q u^{q-1}(\nabla u - \nabla v) + q \nabla v( u^{q-1} - v^{q-1} ), \end{aligned}$$

so that

$$\begin{aligned} \int _{\Omega \times \{t_2\}}&G_\delta (u^q - v^q)\,\textrm{d}x- \int _{\Omega \times \{t_1\}} G_\delta (u^q - v^q)\,\textrm{d}x\\&\le -\frac{q}{\delta } \iint _{\Omega _{\delta }} u^{q-1} \underbrace{\left( |\nabla u|^{p-2}\nabla u - |\nabla v|^{p-2}\nabla v \right) \cdot \left( \nabla u - \nabla v\right) }_{\ge 0}\,\textrm{d}x\textrm{d}t\\&\quad - \frac{q}{\delta }\iint _{\Omega _{\delta }} \big (u^{q-1} - v^{q-1}\big )\left( |\nabla u|^{p-2}\nabla u - |\nabla v|^{p-2}\nabla v \right) \cdot \nabla v\,\textrm{d}x\textrm{d}t\\&\le - \frac{q}{\delta }\iint _{\Omega _{\delta }} \big (u^{q-1} - v^{q-1}\big )\left( |\nabla u|^{p-2}\nabla u - |\nabla v|^{p-2}\nabla v \right) \cdot \nabla v\,\textrm{d}x\textrm{d}t. \end{aligned}$$

In view of Lemma 2.3 and assumption (3.3), we obtain in the set $\Omega _\delta$ the following estimate

$$\begin{aligned} 0 < u^{q-1} - v^{q-1} = \left| u^{q \frac{q-1}{q}} - v^{q\frac{q-1}{q}}\right| \le c(q) (u^q+v^q)^{-\frac{1}{q}}|u^q-v^q| \le \frac{c(q)}{\epsilon }\, \delta . \end{aligned}$$

This yields

$$\begin{aligned} \int _{\Omega \times \{t_2\}}&G_\delta (u^q - v^q) \,\textrm{d}x\, - \int _{\Omega \times \{t_1\}} G_\delta (u^q- v^q )\,\textrm{d}x\\&\le \frac{c(q)}{\epsilon } \iint _{\Omega _{\delta }} \left| |\nabla u|^{p-2}\nabla u - |\nabla v|^{p-2}\nabla v \right| |\nabla v|\,\textrm{d}x\textrm{d}t. \end{aligned}$$

We now pass to the limit $\delta \downarrow 0$ on both sides. The integral on the right-hand side vanishes, since $| \Omega _{\delta }| \rightarrow 0$ as $\delta \downarrow 0$. Therefore, we obtain

$$\begin{aligned} \int _{\Omega \times \{t_2\}} (u^q - v^q )_+\,\textrm{d}x\le \int _{\Omega \times \{t_1\}} (u^q - v^q )_+\,\textrm{d}x, \end{aligned}$$

which finishes the proof of the proposition. $\square$

We are now in the position to prove our first main result.

#### Proof of Theorem 1.1

The assumptions of the theorem ensure that there exists $\epsilon >0$ such that $v\ge \epsilon$ on $\partial \Omega \times (0,T)$.

We first consider the case $0<q\le 1$. We choose $\kappa \in (0,\epsilon ]$ and define

$$\begin{aligned} u_\kappa :=\max \left\{ u,\kappa \right\} . \end{aligned}$$

Due to Lemma 2.4, we know that $u_\kappa$ is a weak sub-solution to (1.1) in $\Omega _T$. Moreover, in view of assumptions (1.2) and (1.3) we have $(u_\kappa -v)_+\in L^p(0,T;W^{1,p}_0(\Omega ))$. Therefore, we may apply Proposition 3.2 to $u_\kappa$ and *v* to conclude that

$$\begin{aligned} \displaystyle \int _{\Omega \times \{t_2\}} \big (u_\kappa ^q - v^q \big )_+\,\textrm{d}x\le \displaystyle \int _{\Omega \times \{t_1\}} \big (u_\kappa ^q - v^q \big )_+\,\textrm{d}x\end{aligned}$$

for every $0\le t_1<t_2\le T$. Letting $\kappa \downarrow 0$ finishes the proof for $0<q\le 1$.

Next, we consider the case $q\ge 1$. By assumption, there exists a constant $M>0$ such that $u\le M$ on $\partial \Omega \times (0,T)$. For $\kappa \in (0,\epsilon ]$, we now define

3.9 $$\begin{aligned} u_\kappa :=\max \left\{ u,\kappa \right\} \quad \text{ and }\quad v_M :=\min \left\{ v,M \right\} . \end{aligned}$$

Thanks to Lemma 2.4, we know that $u_\kappa$ is a weak sub-solution and $v_M$ is a bounded weak super-solution to (1.1) in $\Omega _T$. Moreover, in view of (1.2) and (1.3) we have $(u_\kappa -v_M)_+\in L^p(0,T;W^{1,p}_0(\Omega ))$. As before, we apply Proposition 3.2 to $u_\kappa$ and $v_M$ to conclude that

$$\begin{aligned} \displaystyle \int _{\Omega \times \{t_2\}} \big (u_\kappa ^q - v_M^q \big )_+\,\textrm{d}x\le \displaystyle \int _{\Omega \times \{t_1\}} \big (u_\kappa ^q - v_M^q \big )_+\,\textrm{d}x\end{aligned}$$

for every $0\le t_1<t_2\le T$. The claim now follows by letting $\kappa \downarrow 0$ and $M\rightarrow \infty$. $\square$

Theorem 1.2 is an immediate consequence of Theorem 1.1.

#### Proof of Theorem 1.2

Applying Theorem 1.1 with the choice $t_1=0$, we obtain

$$\begin{aligned} \int _{\Omega \times \{t\}} (u^q - v^q )_+\,\textrm{d}x\le \int _{\Omega \times \{0\}} (u^q - v^q )_+\,\textrm{d}x\end{aligned}$$

for any $t\in (0,T)$. Since $u(\cdot ,0)^q\le v(\cdot ,0)^q$ a.e. in $\Omega$, the right-hand side of the preceding inequality vanishes, so that

$$\begin{aligned} \int _{\Omega \times \{t\}} (u^q - v^q)_+\,\textrm{d}x\le 0 \end{aligned}$$

for any $t\in (0,T)$. This yields $(u^q - v^q)_+ = 0$ a.e. in $\Omega$ for any $t\in (0,T)$, which implies the desired inequality. $\square$

### Comparison principles in a local setting

The comparison principles in Theorems 1.1 and 1.2 require an upper bound of the weak sub-solution on the lateral boundary of $\Omega _T$ in the case $q>1$. However, some typical applications of the comparison principle are in a local setting. For instance, two solutions shall be compared on a compactly contained subset of $\Omega _T$. For certain ranges of exponents, it is known that weak sub-solutions are locally bounded. We summarize these results in the following remark.

#### Remark 3.3

Let $q>0$ and $p>1$ satisfy either $0<q\le p-1$, or $0<p-1<q<\frac{n(p-1)+p}{(n-p)_+}$. Then, any non-negative weak sub-solution *u* of (1.1) in $\Omega _T$ is locally bounded.

The results are scattered in the literature for different ranges of exponents. A natural classification is the following one:

- $0<q<p-1$ (slow diffusion case), cf. [19] or [8, Theorem 4.1];
- $0<q=p-1$ (homogeneous case), cf. [19] or [22, Lemma 5.1];
- $0<p-1<q<\frac{n(p-1)+p}{(n-p)_+}$ (fast diffusion case), cf. [19] or [6, Theorem 1.3].

This information allows to omit the boundedness assumption in the comparison principle in a local setting.

#### Corollary 3.4

Let $q>0$ and $p>1$ satisfy either $0<q\le p-1$, or $0<p-1<q<\frac{n(p-1)+p}{(n-p)_+}$, and let *u* be a non-negative local weak sub-solution and *v* a non-negative local weak super-solution of (1.1) in $\Omega _T$. Further, let $K \Subset \Omega$ and $0<t_1<t_2<T$ and suppose that

$$\begin{aligned} \mathop {\mathrm {ess\,inf}}\limits \limits _{\partial K\times (t_1,t_2)}v>0 \end{aligned}$$

holds. If

$$\begin{aligned} u\le v \qquad \text{ on } \partial _p \big (K\times (t_1,t_2) \big ), \end{aligned}$$

then we have

$$\begin{aligned} u\le v\qquad \text{ a.e. } \text{ in } K\times (t_1,t_2). \end{aligned}$$

#### Proof

In view of Remark 3.3, we know that under the present assumptions *u* is locally bounded in $\Omega _T$. Hence, *u* is bounded in ${\overline{K}}\times [t_1,t_2]$ and *u* is a non-negative weak sub-solution and *v* a non-negative weak super-solution of (1.1) in $K\times (t_1,t_2)$. This allows to apply Theorem 1.2 to *u* and *v* on the parabolic cylinder $K\times (t_1,t_2)$ with the result that $u\le v$ a.e. in $K\times (t_1,t_2)$. $\square$

Corollary 3.4 still requires the super-solution to be strictly positive on the lateral boundary of the considered subcylinder. We are able to omit this assumption in the smaller range of exponents $p-1< q < \frac{n(p-1)}{(n-p)_+}$. In fact, in this case there holds a Harnack inequality without time gap [6, 22]. This allows to prove the comparison principle for non-negative weak solutions stated in Theorem 1.3 without a lower bound on the lateral boundary data.

#### Proof of Theorem 1.3

From [22] in the case $q=p-1$, respectively, [6, Theorem 1.11] in the case $p-1< q < \frac{n(p-1)}{(n-p)_+}$ we know that for any $t\in [t_1,t_2]$ either $u(\cdot ,t)> 0$ or $u(\cdot ,t)\equiv 0$ in ${\overline{K}}$. Moreover, from [6, 7, 24] we know that *u* and *v* are Hölder continuous in ${\overline{K}}\times [t_1,t_2]$. We now let

$$\begin{aligned} \tau _o:= \sup \big \{t\in [t_1,t_2]: u(\cdot ,t)\le v(\cdot ,t) \text{ in } {\overline{K}}\big \}. \end{aligned}$$

Note that $u(\cdot ,\tau _o)\le v(\cdot ,\tau _o)$ in ${\overline{K}}$ by the continuity of *u* and *v* if $\tau _o>t_1$, respectively, by the initial condition $u(\cdot ,t_1)\le v(\cdot ,t_1)$ if $\tau _o=t_1$.

We claim that $\tau _o<t_2$. As explained above, we either have $u(\cdot ,\tau _o)> 0$ or $u(\cdot ,\tau _o)\equiv 0$ in ${\overline{K}}$. In the former case, there exist $\epsilon >0$ and $0<\delta \le t_2-\tau _o$ such that $u\ge \epsilon$ in $\overline{K}\times [\tau _o,\tau _o+\delta ]$. Moreover, we have $u\le v$ on $\partial _p (K\times (\tau _o,\tau _o+\delta ))$. This allows to apply Theorem 1.2 to conclude that $u\le v$ in $K\times (\tau _o,\tau _o+\delta )$, contradicting $\tau _o<t_2$.

In the latter case, where $u(\cdot ,\tau _o)\equiv 0$ in $\overline{K}$, there exists $\tau _1\in (\tau _o,t_2]$ such that $u>0$ in ${\overline{K}}\times (\tau _o,\tau _1]$. Moreover, there exist $\epsilon >0$ and $0<\delta <\tau _1-\tau _o$ such that $u\ge \epsilon$ in $\overline{K}\times [\tau _o+\delta ,\tau _1]$. Since $v\ge u\ge \epsilon$ on $\partial K\times [\tau _o+\delta ,\tau _1]$, Theorem 1.1 implies

$$\begin{aligned} \int _{K\times \{t\}}\left( u^q - v^q\right) _+\,\textrm{d}x\le \int _{K\times \{\tau _o+\delta \}}\left( u^q - v^q\right) _+\,\textrm{d}x\end{aligned}$$

for any $\tau _o+\delta \le t\le t_2$. Letting $\delta \downarrow 0$ in the inequality above, the integral on the right hand side vanishes, since *u* and *v* are continuous and $u(\cdot ,\tau _o)=0$ in $\overline{K}$. This, however, implies $u\le v$ in $\overline{K}\times [\tau _o,\tau _1]$, again contradicting $\tau _o<t_2$.

Hence, we have $\tau _o=t_2$, which implies $u\le v$ in $K\times (t_1,t_2)$ as claimed. $\square$

## General structures

In this section, we present some generalizations under which the statements of the comparison principles continue to hold.

### Inhomogeneous equations

The first generalization concerns the presence of a right-hand side. Instead of (1.1), we now consider its inhomogeneous variant

4.1 $$\begin{aligned} \partial _t u^q - \Delta _p u =f\qquad \text{ in } \Omega _T \end{aligned}$$

for some

$$\begin{aligned} f\in L^{{{\tilde{p}}}'}(\Omega _T), \end{aligned}$$

where

$$\begin{aligned} \tilde{p} := \displaystyle \max \{p,q+1\} \end{aligned}$$

and ${{\tilde{p}}}'=\frac{{\tilde{p}}}{{\tilde{p}}-1}$ denotes the Hölder conjugate of ${\tilde{p}}$.

#### Definition 4.1

A non-negative measurable function $u:\Omega _T\rightarrow \mathbb {R}_{\ge 0}$ in the class

$$\begin{aligned} u\in C\big ([0,T];L^{q+1}(\Omega ) \big ) \cap L^{p}\big (0,T;W^{1,p}(\Omega )\big ) \end{aligned}$$

is a weak sub(super)-solution of (4.1) if

4.2 $$\begin{aligned} \iint _{\Omega \times (t_1,t_2)}\big [-u^q\partial _t\phi + |\nabla u|^{p-2}\nabla u \cdot \nabla \phi \big ]\,\textrm{d}x\textrm{d}t\le (\ge )\, \iint _{\Omega \times (t_1,t_2)}f\phi \,\textrm{d}x\textrm{d}t \end{aligned}$$

for any non-negative function

$$\begin{aligned} \phi \in W^{1,q+1}_{0}\left( 0,T;L^{q+1}(\Omega )\right) \cap L^p\big (0,T;W^{1,p}_0(\Omega )\big ). \end{aligned}$$

A function *u* is a non-negative weak solution of (4.1) if it is both, a weak sub-solution and a weak super-solution.

The next lemma is a generalization of Lemma 2.4 for the inhomogeneous case.

#### Lemma 4.2

Let $q>0$, $p>1$ and *u* be a non-negative weak sub-solution of (4.1) in the sense of Definition 4.1. Then, for any $\kappa >0$ the function $\max \{u,\kappa \}$ is a weak sub-solution ofSimilarly, if *v* is a non-negative weak super-solution of (4.1), then for any $M>0$ also $\min \{v,M\}$ is a weak super-solution of

#### Proof

We only treat the first part of the Lemma concerning sub-solutions, since the second one follows with a similar reasoning. We argue exactly as in the proof of Lemma 2.4 with the only exception that we have to treat the additional term

$$\begin{aligned} \iint _{\Omega _T}f\phi \,\textrm{d}x\textrm{d}t\end{aligned}$$

that appears on the right-hand side of (2.6). Inserting the test-function

$$\begin{aligned} \phi = \eta \phi _h, \quad \text{ where } \phi _h:= \frac{(\llbracket u\rrbracket _{{\bar{h}}}-\kappa )_+}{(\llbracket u\rrbracket _{{\bar{h}}}-\kappa )_++\mu }, \end{aligned}$$

as defined in the proof of Lemma 2.4, passing first to the limit $h \downarrow 0$ and afterward to the limit $\mu \downarrow 0$, the integral converges to

$$\begin{aligned} \iint _{\Omega _T\cap \{u>\kappa \}}f\eta \,\textrm{d}x\textrm{d}t. \end{aligned}$$

As in the proof of Lemma 2.4, we now use an approximation argument in order to replace $\eta$ by an arbitrary testing function

$$\begin{aligned} \phi \in W^{1,q+1}_{0}\big (0,T;L^{q+1}(\Omega )\big ) \cap L^p\big (0,T;W^{1,p}_0(\Omega )\big ). \end{aligned}$$

This proves that $\max \{u,k\}$ is a sub-solution as claimed. $\square$

In the inhomogeneous case, we obtain the following variant of Theorem 1.1.

#### Corollary 4.3

Let $$p>1$$, $$q>0$$ and

$$\begin{aligned} f_1,\,f_2 \in L^{{{\tilde{p}}}'}(\Omega _T). \end{aligned}$$

Further, let *u* be a non-negative weak sub-solution of

$$\begin{aligned} \partial _t u^q - \Delta _p u = f_1\qquad \text{ in } \Omega _T \end{aligned}$$

and *v* be a weak non-negative super-solution of

$$\begin{aligned} \partial _t v^q - \Delta _p v = f_2\qquad \text{ in } \Omega _T \end{aligned}$$

satisfying

4.3 $$\begin{aligned} \mathop {\mathrm {ess\,inf}}\limits \limits _{\partial \Omega \times (0,T)}v>0 \qquad \text{ and } \qquad \mathop {\mathrm {ess\,sup}}\limits \limits _{\partial \Omega \times (0,T)}u<\infty \text{ if } q>1. \end{aligned}$$

If

$$\begin{aligned} u\le v \quad \text{ on } \partial \Omega \times (0,T), \end{aligned}$$

then the following inequality holds

$$\begin{aligned} \int _{\Omega \times \{t_2\}}(u^q-v^q)_+\,\textrm{d}x\le \int _{\Omega \times \{t_1\}}(u^q-v^q)_+\,\textrm{d}x+ \iint _{\Omega \times (t_1,t_2) \cap \left\{ v < u \right\} }(f_1-f_2)\,\textrm{d}x\textrm{d}t\end{aligned}$$

for every $$0\le t_1<t_2\le T$$.

#### Proof

The claimed inequality may be shown in a similar way as Theorem 1.1 taking also into account the additional terms containing $f_1$ and $f_2$. In the following, we will explain in the case $q\ge 1$ how these terms are dealt with. We choose $0<\kappa<\epsilon \le M<\infty$ and define $u_\kappa =\max \{u,\kappa \}$ and $v_M=\min \{v,M\}$ as in the proof of Theorem 1.1. Instead of Lemma 2.4, we now apply Lemma 4.2 to infer that $u_\kappa$ is a weak sub-solution toand $v_M$ is a weak super-solution toSubsequently, we need a variant of Proposition 3.2 for inhomogeneous equations. Performing the same arguments as in the proof of Proposition 3.2, we obtain in inequality (3.5) the additional termon the right-hand side. Passing to the limit $h\downarrow 0$ and $\delta \downarrow 0$, we obtain instead of (3.4) the following inequality:Note that $\{u_\kappa>v_M\}=\{u>v\}$, since $\kappa <M$. Finally, passing to the limits $\kappa \downarrow 0$ and $M\rightarrow \infty$, yields the claimed inequality for $q\ge 1$. The modifications in the case $0<q\le 1$ are similar. $\square$

In the case $f_1=f_2$, integral term on the right-hand side vanishes and therefore, we obtain the following variant of Theorem 1.2, which immediately follows from Corollary 4.3.

#### Corollary 4.4

Let $$q>0$$, $$p>1$$ and

$$\begin{aligned} f\in L^{{{\tilde{p}}}'}(\Omega _T) \end{aligned}$$

and *u* be a non-negative weak sub-solution and *v* a non-negative weak super-solution of (4.1) in $\Omega _T$ satisfying (4.3). If

$$\begin{aligned} u\le v \qquad \text{ on } \partial _p \Omega _T, \end{aligned}$$

then we have

$$\begin{aligned} u\le v\qquad \text{ a.e. } \text{ in } \Omega _T. \end{aligned}$$

### General coefficients

Instead of the model equation (1.1), respectively, (4.1), one may consider some more general doubly nonlinear equations. More precisely, instead of the *p*-Laplacian operator we consider vector fields

$$\begin{aligned} A(x,t,u,\xi ):\mathbb {R}^n\times \mathbb {R}_+\times \mathbb {R}_+\times \mathbb {R}^n \rightarrow \mathbb {R}^n \end{aligned}$$

and the associated doubly nonlinear equation

4.4 $$\begin{aligned} \partial _t u^q - {{\,\textrm{div}\,}}A(x,t,u,\nabla u) = f \qquad \text{ in } \Omega _T, \end{aligned}$$

where $q>0$. The vector field *A* is supposed to be a Carathéodory function, which means

$$\begin{aligned}&(x,t)\mapsto A(x,t,u,\xi )\,\,\text{ is } \text{ measurable } \text{ for } \text{ every } (u,\xi ) \in \mathbb {R}\times \mathbb {R}^n, \\&(u,\xi )\mapsto A(x,t,u,\xi )\,\, \text{ is } \text{ continuous } \text{ for } \text{ almost } \text{ every } (x,t) \in \Omega _T, \end{aligned}$$

and further to satisfy the following conditions

4.5 $$\begin{aligned} \left\{ \begin{array}{l} A(x,t,u,0) = 0 \\ \big ( A(x,t,u,\xi ) - A(x,t,u,\eta ) \big ) \cdot \left( \xi - \eta \right) \ge 0 \\ \left| A(x,t,u,\xi )\right| \le C\big (1 + \left| \xi \right| ^{p-1} \big ) \\ \left| A(x,t,u,\xi ) - A(x,t,v,\xi )\right| \le L\left| u-v \right| \big (1 + \left| \xi \right| ^{p-1} \big ) \end{array} \right. \end{aligned}$$

for a.e. $(x,t)\in \Omega _T$ and any $u,v\in \mathbb {R}$, and any $\xi ,\eta \in \mathbb {R}^n$, where $p>1$ and *C* and *L* denote positive constants.

#### Definition 4.5

A non-negative measurable function $u:\Omega _T\rightarrow \mathbb {R}_{\ge 0}$ in the class

$$\begin{aligned} u\in C\big (0,T;L^{q+1}(\Omega ) \big ) \cap L^{p}\big (0,T;W^{1,p}(\Omega )\big ) \end{aligned}$$

is a non-negative weak sub(super)-solution of (4.4) if

4.6 $$\begin{aligned} \iint _{\Omega \times (t_1,t_2)}\big [-u^q \partial _t\phi + A(x,t,u,\nabla u) \cdot \nabla \phi \big ]\,\textrm{d}x\textrm{d}t\le (\ge )\, \iint _{\Omega \times (t_1,t_2)}f\phi \,\textrm{d}x\textrm{d}t \end{aligned}$$

for any non-negative function

$$\begin{aligned} \phi \in W^{1,q+1}_{0}\left( 0,T;L^{q+1}(\Omega )\right) \cap L^p\big (0,T;W^{1,p}_0(\Omega )\big ). \end{aligned}$$

A function *u* is a non-negative weak solution of (4.4) if it is both, a weak sub-solution and a weak super-solution.

Due to the structure condition (4.5)_3_ and the definition of ${\tilde{p}}'$, both integrals in (4.6) are finite. Moreover, we mention that the assumed continuity in time of weak sub/super-solutions in the sense of Definition 4.5 is not restrictive, see [6, Proposition 4.9]. Note that the doubly nonlinear equation (1.1) is a special case of (4.4), since $A(x,t,u,\nabla u) = A(\nabla u) = \left| \nabla u \right| ^{p-2} \nabla u$ satisfies hypothesis (4.5).

The subsequent Lemma is a variant of Lemma 2.4 for the more general equations considered above.

#### Lemma 4.6

Let $q>0$, $p>1$ and *u* be a non-negative weak sub-solution of (4.4) in the sense of Definition 4.5. Then, for any $\kappa >0$ the function $\max \{u,\kappa \}$ is a weak sub-solution ofSimilarly, if *v* is a non-negative local weak super-solution of (4.4), then for any $M>0$ also $\min \{v,M\}$ is a local weak super-solution of

#### Proof

The proof is similar to the one of Lemma 2.4, respectively, Lemma 4.2 for the model pdes (1.1) and (4.1). The proof for the case $f\equiv 0$ a.e. in $\Omega _T$ can be found in [6, Proposition 4.7]. Note that assumption (4.5)_1_ is needed here in order to avoid a multiplicative factor , respectively,  of the vector field *A*, see [6, Remark 4.8]. Moreover, the right-hand side *f* can be treated as in the proof of Lemma 4.2. $\square$

The following Corollary illustrates another version of Theorem 1.1, which is the most general comparison principle in this paper.

#### Corollary 4.7

Let $$q>0$$, $$p>1$$ and

$$\begin{aligned} f_1,\,f_2 \in L^{{{\tilde{p}}}'}(\Omega _T) \end{aligned}$$

Further, let *u* be a non-negative weak sub-solution of

$$\begin{aligned} \partial _t u^q - {{\,\textrm{div}\,}}{\left( A(x,t,u,\nabla u) \right) } = f_1 \qquad \text{ in } \Omega _T \end{aligned}$$

and *v* be a non-negative weak super-solution of

$$\begin{aligned} \partial _t v^q - {{\,\textrm{div}\,}}{\left( A(x,t,u,\nabla v) \right) } = f_2 \qquad \text{ in } \Omega _T \end{aligned}$$

satisfying

4.7 $$\begin{aligned} \mathop {\mathrm {ess\,inf}}\limits \limits _{\partial \Omega \times (0,T)}v>0 \qquad \text{ and } \qquad \mathop {\mathrm {ess\,sup}}\limits \limits _{\partial \Omega \times (0,T)}u<\infty \text{ if } q>1. \end{aligned}$$

If

$$\begin{aligned} u\le v \quad \text{ on } \partial \Omega \times (0,T), \end{aligned}$$

then the following inequality holds

$$\begin{aligned} \displaystyle \int _{\Omega \times \{t_2\}}(u^q-v^q)_+\,\textrm{d}x\le \displaystyle \int _{\Omega \times \{t_1\}}(u^q-v^q)_+\,\textrm{d}x+ \iint _{\Omega \times (t_1,t_2) \cap \left\{ v < u \right\} }(f_1-f_2)\,\textrm{d}x\textrm{d}t\end{aligned}$$

for every $$0\le t_1<t_2\le T$$.

#### Proof

The proof can be achieved by similar arguments as in Theorem 1.1, taking into account the more general vector field *A*. The right-hand side can be treated exactly as in Corollary 4.3. Therefore, we only explain the arguments needed to treat the vector field *A* and omit the terms containing the functions $f_1$ and $f_2$. In the case $q\ge 1$, a similar approach to the proof of Theorem 1.1 leads us to the following version of (3.7)

$$\begin{aligned} \int _{\Omega \times \{t_2\}}&G_\delta \left( u_\kappa ^q - v_M^q \right) \,\textrm{d}x- \int _{\Omega \times \{t_1\}} G_\delta \left( u_\kappa ^q - v_M^q \right) \,\textrm{d}x\\&\le - \frac{1}{\delta } \iint _{\Omega _{\delta }}\big (A(x,t,u_\kappa ,\nabla u_\kappa ) - A(x,t,v_M,\nabla v_M)\big )\cdot \nabla (u_\kappa ^q - v_M^q) \,\textrm{d}x\textrm{d}t. \end{aligned}$$

Here, $\Omega _\delta$ denotes the set

$$\begin{aligned} \Omega _{\delta }:= \big \{(x,t)\in \Omega \times (t_1,t_2) :\, 0< u_\kappa ^q(x,t) - v_M^q(x,t) < \delta \big \}. \end{aligned}$$

Due to identity (3.8), the right-hand side of the preceding inequality may be re-written as

$$\begin{aligned}&- \frac{1}{\delta } \iint _{\Omega _{\delta }} \big (A(x,t,u_\kappa ,\nabla u_\kappa ) - A(x,t,v_M,\nabla v_M)\big )\cdot \nabla (u_\kappa ^q - v_M^q) \,\textrm{d}x\textrm{d}t\\&\quad = - \frac{1}{\delta } \iint _{\Omega _{\delta }} u_\kappa ^{q-1}\big (A(x,t,u_\kappa ,\nabla u_\kappa ) - A(x,t,v_M,\nabla v_M)\big )\cdot \left( \nabla u_\kappa - \nabla v_M\right) \,\textrm{d}x\textrm{d}t\\&\qquad - \frac{1}{\delta } \iint _{\Omega _{\delta }}q \big (u_\kappa ^{q-1} - v_M^{q-1}\big ) \big (A(x,t,u_\kappa ,\nabla u_\kappa ) - A(x,t,v_M,\nabla v_M)\big )\cdot \nabla v_M \,\textrm{d}x\textrm{d}t. \end{aligned}$$

The second term on the right-hand side of the above identity vanishes in the limit $\delta \downarrow 0$, which follows similarly as in the proof of Theorem 1.1. Therefore, we will concentrate on the first term. Using assumptions (4.5)_2_ and (4.5)_4_ together with the fact that $u_\kappa \ge \kappa$ in $\Omega _T$, we obtain

$$\begin{aligned} - \frac{1}{\delta }&\iint _{\Omega _{\delta }} u_\kappa ^{q-1}\big (A(x,t,u_\kappa ,\nabla u_\kappa ) - A(x,t,v_M,\nabla v_M)\big )\cdot \left( \nabla u_\kappa - \nabla v_M\right) \,\textrm{d}x\textrm{d}t\\&= - \frac{1}{\delta } \iint _{\Omega _{\delta }} u_\kappa ^{q-1} \underbrace{\big (A(x,t,u_\kappa ,\nabla u_\kappa ) - A(x,t,u_\kappa ,\nabla v_M)\big )\cdot \left( \nabla u_\kappa - \nabla v_M\right) }_{\ge 0} \,\textrm{d}x\textrm{d}t\\&\quad - \frac{1}{\delta } \iint _{\Omega _{\delta }} u_\kappa ^{q-1} \big (A(x,t,u_\kappa ,\nabla v_M) - A(x,t,v_M,\nabla v_M)\big )\cdot \left( \nabla u_\kappa - \nabla v_M\right) \,\textrm{d}x\textrm{d}t\\&\le \frac{1}{\delta } \iint _{\Omega _{\delta }} u_\kappa ^{q-1}\left| A(x,t,u_\kappa ,\nabla v_M) - A(x,t,v_M,\nabla v_M)\right| \left| \nabla u_\kappa - \nabla v_M\right| \,\textrm{d}x\textrm{d}t\\&\le \frac{L}{\delta } \iint _{\Omega _{\delta }} u_\kappa ^{q-1}\left| u_\kappa - v_M\right| \big (1+\left| \nabla v_M\right| ^{p-1} \big ) \left| \nabla u_\kappa - \nabla v_M\right| \,\textrm{d}x\textrm{d}t\\&\le L\,c(q,\kappa ) \iint _{\Omega _{\delta }} u_\kappa ^{q-1}\big (1+\left| \nabla v_M\right| ^{p-1} \big ) \left| \nabla u_\kappa - \nabla v_M\right| \,\textrm{d}x\textrm{d}t. \end{aligned}$$

The last integral vanishes in the limit $\delta \downarrow 0$. Finally, letting $\kappa \downarrow 0$ and $M\rightarrow \infty$ finishes the proof in the case $q\ge 1$. Since the case $0<q<1$ is similar, we omit the details. $\square$

The following corollary represents a generalization of Theorem 1.2 for the doubly nonlinear equation (4.4).

#### Corollary 4.8

Let $$q>0$$, $$p>1$$ and

$$\begin{aligned} f\in L^{{{\tilde{p}}}'}(\Omega _T) \end{aligned}$$

and *u* be a non-negative weak sub-solution and *v* a non-negative weak super-solution of (4.4) in $\Omega _T$ satisfying (4.7). If

$$\begin{aligned} u\le v \qquad \text{ on } \partial _p \Omega _T, \end{aligned}$$

then we have

$$\begin{aligned} u\le v\qquad \text{ a.e. } \text{ in } \Omega _T. \end{aligned}$$

#### Remark 4.9

Similar local results as obtained in Sect. 3.2 also hold true for the doubly-nonlinear equation (4.4). Corollary 3.4 still holds true, provided the right-hand side *f* is integrable enough to ensure local boundedness of the sub-solution. Theorem 1.3 continues to hold for homogeneous equations of the more general structure (4.4), i.e., $f\equiv 0$. Note that the main ingredient in the proof is a time insensitive Harnack inequality, which is available also under these more general assumptions; see [6, Theorem 1.10].

## Uniqueness

The comparison principles derived so far imply uniqueness of weak solutions to the associated Cauchy–Dirichlet problem. Since only non-negative weak solutions are considered, the boundary and initial data are assumed to be non-negative as well. Note that due to Corollary 4.3 we are able to also consider a nontrivial right-hand side *f* in the Cauchy–Dirichlet problem.

### Theorem 5.1

Consider the data

$$\begin{aligned} \left\{ \begin{array}{l} f\in L^{{{\tilde{p}}}'}(\Omega _T), \\ g\in L^p\big (0,T;W^{1,p}(\Omega )\big ), \\ u_o\in L^{2}\big (\Omega ,\mathbb {R}_{\ge 0}\big ). \end{array} \right. \end{aligned}$$

Suppose furthermore that $g \ge \epsilon$ for some $\epsilon >0$ and in the case $q>1$ additionally assume that *g* is bounded. Then, there exists a unique non-negative weak solution of the Cauchy–Dirichlet problem

5.1 $$\begin{aligned} {\left\{ \begin{array}{ll} \partial _t u^q - \Delta _p u = f &{} \,\,\text{ in } \Omega \times (0,T),\\ u=g &{} \,\,\text{ on } \partial \Omega \times (0,T), \\ u(\cdot ,0) = u_o &{} \,\,\text{ in } \Omega . \end{array}\right. } \end{aligned}$$

### Proof

The existence of a weak solution can be inferred for instance from [1]. Let $u_1$ and $u_2$ be two non-negative weak solutions of (5.1). Then, we have

$$\begin{aligned} u_1 - u_2 = \left( u_1 - g\right) - \left( u_2-g\right) \in L^p\big (0,T;W^{1,p}_0(\Omega )\big ) \end{aligned}$$

and similarly for the initial datum

$$\begin{aligned} u_1(\cdot ,0) - u_2(\cdot ,0) = 0\qquad \text{ a.e. } \text{ in } \Omega . \end{aligned}$$

Applying Corollary 4.4 twice, we first derive $u_1 \le u_2$ and similarly $u_1 \ge u_2$ a.e. in $\Omega _T$. In turn, this yields $u_1 = u_2$ a.e. in $\Omega _T$. $\square$

A similar uniqueness result for non-negative weak solutions holds for the more general doubly nonlinear equation (4.4). In the proof, Corollary 4.4 has to be replaced by 4.8.

### Theorem 5.2

Let $f,g,u_o$ be as in Theorem 5.1 and suppose that the vector field *A* satisfies the set of assumptions (4.5). Then, there exists a unique non-negative weak solution of the Cauchy–Dirichlet problem

$$\begin{aligned} {\left\{ \begin{array}{ll} \partial _t u^q - {{\,\textrm{div}\,}}A(x,t,u,\nabla u) = f &{} \,\,\text{ in } \Omega \times (0,T),\\ u=g &{} \,\,\text{ on } \partial \Omega \times (0,T), \\ u(\cdot ,0) = u_o &{} \,\,\text{ in } \Omega . \end{array}\right. } \end{aligned}$$

### Remark 5.3

A uniqueness result is also available in the case that the lateral boundary datum *g* vanishes entirely, see [26, 27]. Moreover, in the case $0<p-1 \le q < \frac{n(p-1)}{(n-p)_+}$, Theorem 1.3 ensures local uniqueness of weak solutions without imposing any additional upper or lower bounds.

## Viscosity solutions

In this final section, we will give an application of the comparison principle and show that every weak solution of (1.1) is also a viscosity solution. The result is interesting in itself as existence of a weak solution thus guarantees existence of a viscosity solution. In a similar fashion, we are also able to give a respective result for the homogeneous version of the generalized pde (4.4). Throughout we refer to [3, 11] for the definition and properties of viscosity solutions.

### Definition 6.1

Let $q>0$, $p\ge 2$ and $u:\Omega _T\rightarrow \mathbb {R}_{\ge 0}$ be an upper semi-continuous function. In the case $0<q<1$, we additionally require $u>0$. *u* is a viscosity sub-solution of (1.1) if for any function $\phi \in C^1((0,T); C^2(\Omega ))$ such that $\phi (x_0,t_0) = u(x_0,t_0)$ and $\phi >u$ in a deleted neighborhood of $(x_0,t_0)$, we have

$$\begin{aligned} \partial _t\phi ^q(x_0,t_0)-\Delta _p \phi (x_0,t_0)\le 0. \end{aligned}$$

Similarly, a lower semi-continuous function $u:\Omega _T\rightarrow \mathbb {R}_{\ge 0}$ is a viscosity super-solution of (1.1) if for any function $\phi \in C^1((0,T); C^2(\Omega ))$ such that $\phi (x_0,t_0) = u(x_0,t_0)$ and $\phi <u$ in a deleted neighborhood of $(x_0,t_0)$, we have

$$\begin{aligned} \partial _t\phi ^q(x_0,t_0)-\Delta _p \phi (x_0,t_0)\ge 0. \end{aligned}$$

Finally, a function *u* is a viscosity solution of (1.1) if it is both, a viscosity sub-solution and a viscosity super-solution.

### Remark 6.2

In the case $1<p<2$, the definition of viscosity solution is delicate, since $\Delta _p\phi$ is not well defined for test functions $\phi$ whose gradient vanishes at the touching point; see [21, 28] for more discussion on this topic. For this reason, we restrict ourselves to the case $p\ge 2$.

### Remark 6.3

In the literature, often strict inequalities are used, cf. [9, 21, 28]. Note that viscosity sub/super-solutions may equivalently be defined without strict inequalities of the test functions touching *u* from either below or above. However, it is always possible to obtain strict inequalities by modifying the test-function, which leads to equivalent Definitions.

We will need the following Lemma to prove the result for viscosity solutions afterward. In the theory of viscosity solutions, the stated property usually is referred to as *degenerate ellipticity*, see [11].

### Lemma 6.4

Let $p\ge 2$ and $\phi \in C^2 (\Omega )$ such that $D^2 \phi \in \mathbb {R}^{n\times n}$ is positive semi-definite. Then, there holds $\Delta _p\phi \ge 0$.

### Proof

Let $x_0 \in \Omega$. In order to simplify notation, we abbreviate $v = \nabla \phi (x_0)$ and $X = D^2 \phi (x_0)$. We compute

$$\begin{aligned} \Delta _p\phi (x_0)&= (p-2)|v|^{p-4} \left( X v\cdot v\right) + |v|^{p-2}{{\,\textrm{Tr}\,}}(X){} & {} \\&\ge |v|^{p-2}\left( -|v|^{-2} \left( X v,v\right) + {{\,\textrm{Tr}\,}}(X) \right){} & {} \\&\ge |v|^{p-2}\bigg (-\max _{i\in \{1,\dots ,n\}}\{\lambda _i \} + \sum \limits _{i=1}^n\lambda _i \bigg ) \ge 0 , \end{aligned}$$

where $\lambda _i$ for $i\in \{1,...,n\}$ denote the eigenvalues of *X* and the estimate

$$\begin{aligned} \frac{\langle Xv,v\rangle }{|v|^2} = \frac{\langle Xv,v\rangle }{\langle v,v\rangle } \le \max \{\lambda _1, ..., \lambda _n\} \end{aligned}$$

was used. $$\square $$

We now state the result about viscosity solutions as an application of the comparison principle in Theorem 1.2. We only show that any weak solution is a viscosity solution in the sense of Definition 6.1. We did not attempt to prove the reverse implication, which is more involved. In the elliptic case, this property has for example been shown in [21], whereas the parabolic *p*-Laplace equation with a more general right hand side has been considered in [28]. In both cases, the weak and viscosity solutions coincide.

### Theorem 6.5

Let $q>0$, $p\ge 2$ and *u* be a bounded non-negative weak solution of

$$\begin{aligned} \partial _t u^q - \Delta _p u = 0 \qquad \text{ in } \Omega _T. \end{aligned}$$

Then, *u* is a viscosity solution of

$$\begin{aligned} \partial _t u^q - \Delta _p u = 0 \qquad \text{ in } \Omega _T \cap \{u>0\}. \end{aligned}$$

If $1\le p-1<q<\frac{n(p-1)}{(n-p)_+}$, then *u* is a viscosity solution of

$$\begin{aligned} \partial _t u^q - \Delta _p u = 0\qquad \text{ in } \Omega _T. \end{aligned}$$

### Proof

Instead of *u*, we consider its upper semi-continuous regularization $u_*$, which is, for locally bounded solutions, uniquely determined and verifies $u = u_*$ a.e. in $\Omega _T$, see [25, Theorem 2.3].

We first show that any upper semi-continuous non-negative weak sub-solution is a viscosity sub-solution in the set $\Omega _T \cap \{u>0\}$. Let $z_0=(x_0,t_0)\in \Omega _T$ with $u(z_0)>0$ and consider a test-function $\phi \in C^1((0,T); C^2(\Omega ))$ with $\phi (z_0)=u(z_0)$ and $\phi >u$ in a deleted neighborhood of $z_0$. Arguing by contradiction, we assume

$$\begin{aligned} \partial _t\phi ^q(z_0)-\Delta _p \phi (z_0)>0. \end{aligned}$$

Since $\phi \in C^1((0,T); C^2(\Omega ))$, this inequality continues to hold in a neighborhood of $z_0$. Hence, we may find $\gamma _0\in (0,1)$ and $\epsilon ,\delta ,\lambda \in (0,1)$ such that

6.1 $$\begin{aligned} \partial _t\phi ^q-\Delta _p \phi \ge \lambda >0 \quad \text{ and }\quad \phi \ge \epsilon \qquad \text{ in } Q_\delta (z_0) \end{aligned}$$

and

6.2 $$\begin{aligned} u\le \gamma _0\phi \qquad \text{ on } \partial _p Q_\delta (z_0), \end{aligned}$$

where $Q_\delta (z_0):= B_\delta (x_0)\times (t_0-\delta ,t_0+\delta )$. The latter is a consequence of the upper semi-continuity of *u*. We abbreviate

$$\begin{aligned} M:= 1+\left\| \partial _t \phi ^q\right\| _{L^\infty (Q_\delta (z_0))} < \infty . \end{aligned}$$

Note that this expression is bounded for any $q>0$, since $\phi \ge \epsilon$ in $Q_\delta (z_0)$. Choosing $\gamma \in [\gamma _0,1)$ large enough to have

$$\begin{aligned} |\gamma ^{q-p+1}-1| \le \frac{\lambda }{M}, \end{aligned}$$

we obtain

$$\begin{aligned} \partial _t(\gamma \phi )^q - \Delta _p(\gamma \phi )&= \gamma ^{p-1}\big [\partial _t \phi ^q - \Delta _p \phi + (\gamma ^{q-p+1} - 1)\partial _t\phi ^q\big ] \\&\ge \gamma ^{p-1}\big [\lambda - |\gamma ^{q-p+1} - 1| M\big ] \ge 0 \end{aligned}$$

in $Q_\delta (z_0)$. Thus, $\gamma \phi \ge \gamma \epsilon >0$ is a classical super-solution and therefore, also a weak super-solution in $Q_\delta (z_0)$. Now, Theorem 1.2 applied with *u* as weak sub-solution and $\gamma \phi$ as weak super-solution yields $u\le \gamma \phi$ in $Q_\delta (z_0)$. Since $0<\gamma <1$, this contradicts $u(x_0,t_0) = \phi (x_0,t_0)>0$. This ensures that *u* is a viscosity sub-solution.

Next, we prove that any lower semi-continuous non-negative weak super-solution is a viscosity super-solution in the set $\Omega _T \cap \{u>0\}$. To this aim, we consider $z_0\in \Omega _T$ with $u(z_0)>0$ and a function $\phi \in C^1((0,T); C^2(\Omega ))$ with $\phi (z_0)<u(z_0)$ and $\phi <u$ in a deleted neighborhood of $z_0$. Again we argue by contradiction and assume

$$\begin{aligned} \partial _t \phi ^q(z_0) -\Delta _p\phi (z_0) <0. \end{aligned}$$

Similarly to before, we find $\gamma _0 >1$ and $\epsilon ,\delta ,\lambda \in (0,1)$ such that

$$\begin{aligned} \partial _t\phi ^q-\Delta _p \phi \le -\lambda <0 \quad \text{ and }\quad \phi \ge \epsilon \qquad \text{ in } Q_\delta (z_0) \end{aligned}$$

and

$$\begin{aligned} u\ge \gamma _0\phi \qquad \text{ on } \partial _p Q_\delta (z_0). \end{aligned}$$

With *M* defined as above, we choose $\gamma \in (1,\gamma _0]$ small enough to have

$$\begin{aligned} |\gamma ^{q-p+1}-1| \le \frac{\lambda }{M}. \end{aligned}$$

In this way, we obtain

$$\begin{aligned} \partial _t(\gamma \phi )^q - \Delta _p(\gamma \phi )&= \gamma ^{p-1}\big [\partial _t \phi ^q - \Delta _p \phi + (\gamma ^{q-p+1} - 1)\partial _t\phi ^q\big ] \\&\le \gamma ^{p-1}\big [-\lambda + |\gamma ^{q-p+1} - 1| M\big ] \le 0 \end{aligned}$$

in $Q_\delta (z_0)$. Now, applying Theorem 1.2 with $\gamma \phi \ge \gamma \epsilon >0$ as weak sub-solution and *u* as weak super-solution we derive a contraction as in the viscosity sub-solution case. This finishes the first part of the Theorem.

To show the second part of the Theorem, we consider $z_0 = (x_0,t_0)\in \Omega _T$. If $u(z_0)>0$, the first part of the theorem applies. Therefore, it remains to consider the case $u(z_0)=0$.

In view of the Harnack inequality from [6, Theorem 1.11], we have $u(\cdot ,t_0)=0$ a.e. in $\Omega$.

We first consider some test function $\phi \in C^1((0,T); C^2(\Omega ))$ such that $\phi (z_0)=u(z_0)$ and $\phi >u$ in a deleted neighborhood of $z_0$. Since *u* and $\phi$ both vanish in $z_0$, it follows that $\phi$ and hence, also $\phi ^q$ attains a minimum there which implies $\partial _t\phi ^q(z_0) =0$ and $\nabla \phi (z_0) = 0$ and $D^2\phi (z_0)$ is positive semi-definite. Now, in view of Lemma 6.4 we obtain the desired inequality

$$\begin{aligned} \partial _t\phi ^q(z_0) - \Delta _p \phi (z_0) = - \Delta _p \phi (z_0) \le 0. \end{aligned}$$

Next, we consider a test function $\phi \in C^1((0,T); C^2(\Omega ))$ such that $\phi (z_0)=u(z_0)$ and $\phi <u$ in a deleted neighborhood of $z_0$. Since $u(\cdot ,t_0)=0$ a.e. in $\Omega$, we have that $\nabla \phi (z_0) = 0$ and $D^2\phi (z_0)$ is negative semi-definite. Moreover, since $q>1$, we have $\partial _t\phi ^q(z_0)=(q-1)\phi ^{q-1}(z_0)\partial _t\phi (z_0)=0,$ so that

$$\begin{aligned} \partial _t\phi ^q(z_0) - \Delta _p \phi (z_0) = - \Delta _p \phi (z_0) \ge 0. \end{aligned}$$

Overall, this shows that *u* is a viscosity solution of (1.1) in $\Omega _T$. $\square$

Note that the Theorem also holds in the range of parameters *p* and *q*, where weak solutions might fail to be locally continuous. This is achieved through the semi-continuous regularization $u_*$ which is defined in the proof. The second part of Theorem 6.5 holds in the whole of $\Omega _T$ due to infinite speed of propagation of weak solutions as shown in [6].

### Remark 6.6

Note that in the second part of Theorem 6.5 we are able to show that any non-negative weak sub-solution is a viscosity sub-solution in $\Omega _T$ for any $q\ge 1$ and $p\ge 2$. The restriction $1\le p-1<q<\frac{n(p-1)}{(n-p)_+}$ is only necessary for the argument ensuring that *u* is a viscosity super-solution.
